# 

**Published:** 1877-01-20

**Authors:** 


ORIGINAL AND SELECTED ARTICLES—
Medical Progress and Medical Journals. By Robert C. Word, M.D. .......... 1
Case of Malformation. Laxative Pill .......... 2
Hospital Reports. Philadelphia Dispensary. Reported by C. C. Vanderbeck, M.D. .... 3
Gelseminum for Dilatation of Cervix .......... 4
Opium Poisoning, Treated by Electricity and Belladonna. By Martin Burke, M.D., of New York City .......... 5
Some Forms of Dyspepsia .......... 6
Eucalyptus as a Disinfectant, Antiseptic and Epidemic Remedy. By A. M. Woodward, M.D., Tunkhannock, Pa .......... 7
Proceedings of the Philadelphia County Medical Society. The Vice-President, Dr. Benjamin Lee, in the Chair .......... 8
The Treatment of Albuminuria .......... 9
A Successful Case of Amputation of the Thigh Treated Antiseptically. By Lewis A. Stimson, M.D., Surgeon to Presbyterian Hospital, New York .......... 10
Nerve Stretching in Tetanus .......... 11
Examination of 'Pain-Killers." .......... 12
ABSTRACTS AND GLEANINGS—
Epilepsy, 12. Post Partem Hemorrhage, Atropia as an Antidote to Hydrocyanic Acid, Bromohydrate of Quinine, Hip Joint Amputation, 13. Hyposulphite of Soda, Salicylate of Soda, Visical Disturbances from Diptheria, Scarlatinal Albuminaria, Petroleum in Cutaneous Affections, 14. Salicylate of Soda as an Antipyretic, Acute Rheumatism Treated by Salicin, Chronic Dysentery, Cured by a Topical Treatment, Torsion, Its Advantages over Ligature in Arresting Hemorrhage, 15, Lister's Antiseptic Method in Ovariotomy, 16. India-Rubber Solution in Psoriasis, Dropsical Effusions, Insect Destroyer, 17. Nasal Catarrh, Exophthalmic Goitre, 18. The Internal Administration of Chloroform, Poisoning by Œnanthe Crocata. Simple Method of Extracting Foreign bodies from the Œsophagus, 19. Warm Water Injections in Dysentery, Bromide of Potassium as a Caustic, Concentrated Solution of Salicylic Acid, 20. Jaborandi as a Calactagogue, Treatment of Orcnitis, Method of Bandaging the Breast, 21. Diseases of the Nose Treated by Medicated Bougies, Solution of Phosphorus in Glycerine, Boldo, Absorption of Iodine by the Skin, Quinine in India, Intermittent Fever, Coffee Leaf, 22.
PRESCRIPTIONS AND FORMULÆ—Syrupus Matico et Cort. Granati, Velpeau's Diarrhœa Mixture (The Pharm.) Haarlem Oil (The Pharm.) "Carbolate of Iodine," 23.
PRACTICAL HINTS.—23.
SCIENTIFIC ITEMS.—24.
EDITORIAL AND MISCELLANEOUS—
Our Journal and its Objects .......... 25
To our Readers—The State Board of Health—The Louisville Embroglio—Arundel Eye-Glasses .......... 26
Specimen Copies—To Physicians and Druggists—Medical Muss at Louisville—New Books .......... 27
Receipts .......... 28

